# Low‐intensity pulsed ultrasound inhibits VEGFA expression in chondrocytes and protects against cartilage degeneration in experimental osteoarthritis

**DOI:** 10.1002/2211-5463.12801

**Published:** 2020-02-05

**Authors:** Mengtong Guan, Ying Zhu, Bo Liao, Qiaoyan Tan, Huabing Qi, Bin Zhang, Junlan Huang, Xiaolan Du, Dingqun Bai

**Affiliations:** ^1^ Department of Rehabilitation Medicine The First Affiliated Hospital of Chongqing Medical University China; ^2^ Department of Rehabilitation Medicine Center of Bone Metabolism and Repair State Key Laboratory of Trauma, Burns and Combined Injury Trauma Center Research Institute of Surgery Daping Hospital Army Medical University (Third Military Medical University) Chongqing China

**Keywords:** articular cartilage, low‐intensity pulsed ultrasound, osteoarthritis, vascular endothelium growth factor A

## Abstract

Low‐intensity pulsed ultrasound (LIPUS), a noninvasive physical therapy, was recently demonstrated to be an effective treatment for osteoarthritis (OA). Vascular endothelium growth factor A (VEGFA) has been found to be upregulated in the articular cartilage, synovium and subchondral bone of OA patients, leading to cartilage degeneration, synovitis and osteophyte formation. However, the functions and mechanisms of LIPUS in regulating chondrocyte‐derived VEGFA expression are still unclear. In this study, we investigated whether LIPUS attenuated OA progression by (a) decreasing the percentage of VEGFA‐positive cells in mouse articular cartilage destabilised through medial meniscus surgery and (b) relieving interleukin‐1β‐induced VEGFA expression in mouse primary chondrocytes. However, this function was negated by a p38 mitogen‐activated protein kinase (p38 MAPK) inhibitor. In addition, we found that LIPUS ameliorated VEGFA‐mediated disorders in cartilage extracellular matrix metabolism and chondrocyte hypertrophy during OA development. In conclusion, our data indicate a novel effect of LIPUS in regulating the expression of osteoarthritic chondrocyte‐derived VEGFA through the suppression of p38 MAPK activity.

## Graphical abstract

Low‐intensity pulsed ultrasound (LIPUS), a noninvasive physical therapy, was recently demonstrated as an effective treatment for osteoarthritis (OA). LIPUS protects articular cartilage from degeneration in experimental OA. In addition, LIPUS ameliorated disorders in cartilage extracellular matrix metabolism and chondrocyte hypertrophy by inhibiting vascular endothelium growth factor A, and this effect is mainly exerted through the inhibition of p38 MAPK signalling.

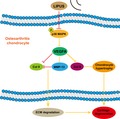

**Abbreviations**
DMM: destabilisation of the medial meniscus
ECM: extracellular matrix
IL‐1β: interleukin‐1β
LIPUS: low‐intensity pulsed ultrasound
MMP‐13: metalloproteinase‐13
OA: osteoarthritis
p38 MAPK: p38 mitogen‐activated protein kinase
VEGFA: vascular endothelium growth factor A

Osteoarthritis (OA) is the most common joint disease, which affects approximately 40% of the world population aged > 70 years 1. OA is pathologically characterised by articular cartilage degeneration, synovial inflammation, subchondral bone remodelling and osteophyte formation 2. The main clinical manifestations of OA patients are joint swelling and pain, which markedly affect the patients’ quality of life 3. However, due to the complicated pathogenesis of OA, the current therapies focus on pain control or total knee replacement 4. Therefore, developing noninvasive methods of OA treatment would be more ideal for patients in the future.

Low‐intensity pulsed ultrasound (LIPUS) is a noninvasive and safe physical therapy 5, which has been widely used in promoting the healing of fresh bone fracture and nonunited fracture 6, 7, 8. Recent clinical trials and basic researches initially confirmed that LIPUS can alleviate OA progression while reducing joint cartilage damage and relieving joint pain 9, 10, 11, 12. A further study demonstrated that LIPUS reduces cartilage damage by promoting chondrocyte proliferation and cartilage matrix secretion 12, 13. However, the comprehensive functions of LIPUS in OA are largely unknown. LIPUS has been clinically used as a supplemental therapy for promoting the healing of bone fracture and wound 14. Moreover, a related study has reported that LIPUS increases the vascular endothelium growth factor A (VEGFA) level of periosteal cells in a mouse femur fracture model, facilitating local angiogenesis and promoting fracture healing 15. However, articular cartilage is a nonvascular tissue, which is composed of extracellular matrix (ECM) and chondrocytes 16. VEGFA is barely expressed by healthy cartilage; however, markedly increased VEGFA expression is related to the severity of OA 17. In addition, injecting VEGFA into both keen and temporomandibular joint induced OA phenotype in mice 18, 19. Correspondingly, another study reported that either deleting VEGFA in Col Π‐Cre lineage cells or intra‐articular injecting anti‐VEGFA antibody attenuated progression of surgically induced OA in mice 20. These findings suggest that controlling VEGFA secretion may help prevent OA escalation and OA‐associated joint deterioration. However, the regulation of VEGFA expression in OA articular chondrocytes by LIPUS is still not explicated.

To address this issue, we investigated whether LIPUS attenuated OA progression by decreasing the percentage of VEGFA‐positive cells in mouse articular cartilage with destabilisation of the medial meniscus (DMM) surgery and relieving interleukin‐1β (IL‐1β)‐induced VEGFA expression in mouse primary chondrocytes.

## Materials and methods

### Isolation and culture of mouse primary chondrocytes

Primary chondrocytes were isolated from the knee articular cartilage of 5‐day‐old mice. Knee joints were first digested by 0.25% trypsinase (Gibco/Life Technologies, Carlsbad, CA, USA) at 37 °C for 15 min, and adjacent muscles, ligaments and bone tissues were removed using a stereomicroscope (Olympus BX51, Tokyo, Japan). Chondrocytes were isolated from the cartilage by additional digestion with 0.1% collagenase Π (Gibco/Life Technologies) overnight at 37 °C in a 5% CO_2_ incubator 21. Cells were seeded in 35‐mm‐diameter dishes and cultured in Dulbecco’s modified Eagle’s medium/F12 (1 : 1) supplemented with 1% penicillin/streptomycin (HyClone, Logan, UT, USA) and 10% FBS (HyClone) and incubated in a humidified atmosphere of 5% CO_2_ at 37 °C. The culture medium was changed every 2 days.

### Cells treated with LIPUS

**Figure 1:**
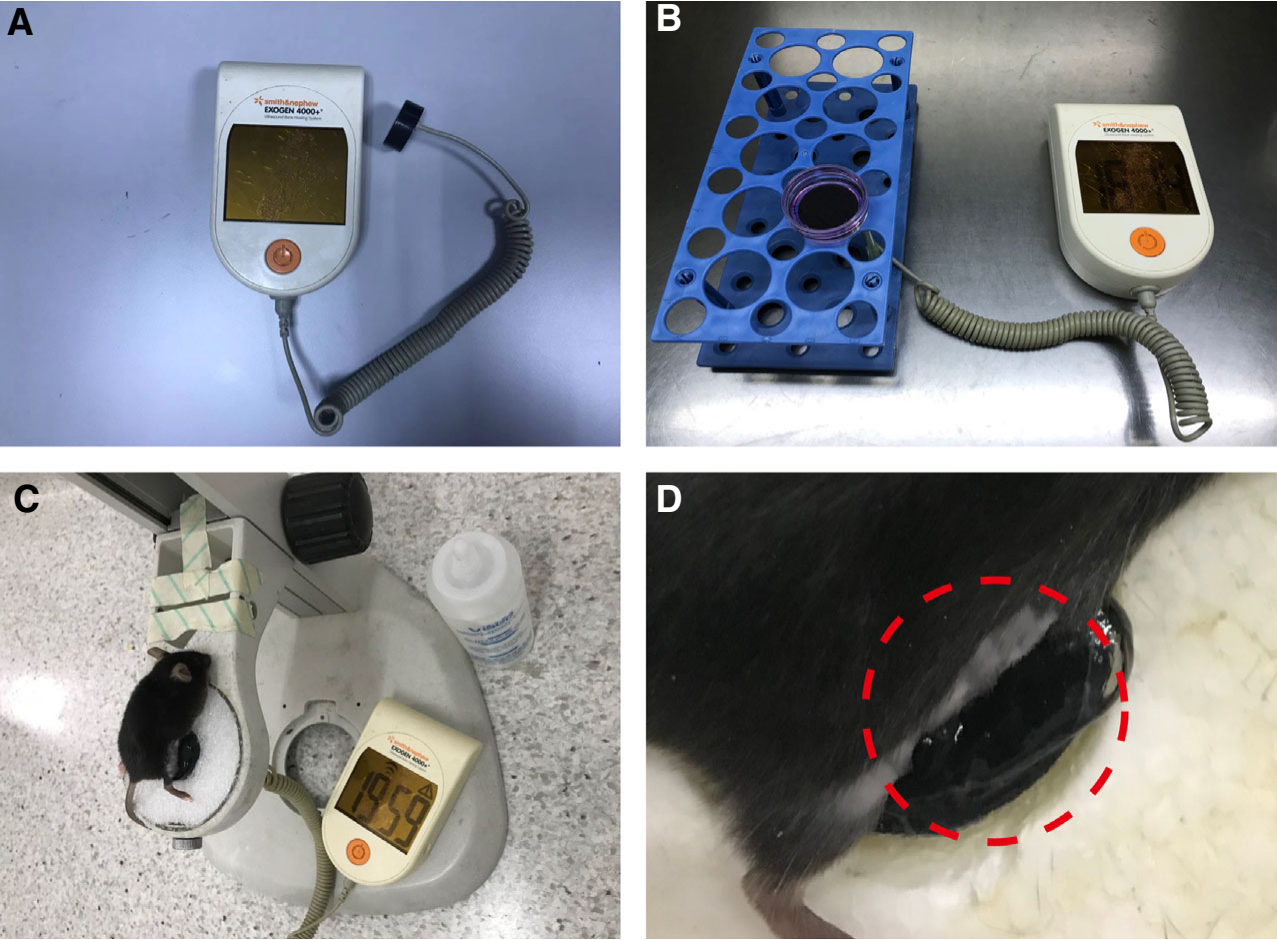
The presentation of LIPUS treatment. The LIPUS system (Exogen 4000; Smith & Nephew) was employed (A). LIPUS treatment of primary chondrocytes *in vitro.* The probe of LIPUS exposure system was placed below the bottom of culture dishes covered by coupling gel (B). LIPUS treatment of mouse knee joints *in vivo*. The right knee joint (the dotted red circle) of mouse was placed on the LIPUS probe which is covered by a layer of coupling agent (C, D).

We treated chondrocytes cultured in 35‐mm‐diameter dishes with LIPUS (Exogen 4000; Smith & Nephew, Jericho, NY, USA) at an average intensity of 30 mW·cm^−2^, frequency of 1.5 MHz, pulse repetition rate of 1 kHz and the on–off ratio of 20% for 20 min (Fig. 1A,B). The stimulations were conducted in a sterile environment at room temperature. The acoustic gel (< 1 mm thick) was used as a coupler between the transducer and cell plate to ensure optimal ultrasound exposure 22.

### Real‐time polymerase chain reaction

Total RNA was isolated from chondrocytes using the TRIzol reagent (Invitrogen, Carlbad, CA, USA) and used to generate cDNA template for real‐time PCR, which was carried out on the Mx3000P system (Exscript, Takara, Japan) using the SYBR Green RT‐PCR Kit (Takara Bio, Shiga, Japan). All samples were measured in triplicate, and the cyclophilin was amplified as an internal control. The primer sequences for RT–PCR are shown in Table 1.

**Table 1:** Sequences of the primers used for RT–PCR.

Target gene	Primer sequences
Cyclophilin	Forward 5 ′ ‐TAGATGGCAAGCATGTCGTGT‐3 ′
	Reverse 5 ′ ‐GTCTTCCCACTTTTTGAGCCG‐3 ′
Collagen II	Forward 5 ′ ‐CTGGTGGAGCAGCAAGAGCAA‐3'
	Reverse 5 ′ ‐CAGTGGACAGTAGACGGAGGAAAG‐3'
VEGFA	Forward 5 ′ ‐GCACATAGAGAGAATGAGCTTCC‐3 ′
	Reverse 5 ′ ‐CTCCGCTCTGAACAAGGCT‐3 ′
MMP‐13	Forward 5 ′ ‐CAGTTGACAGGCTCCGAGAA‐3 ′
	Reverse 5 ′ ‐CGTGTGCCAGAAGACCAGAA‐3 ′
Collagen X	Forward 5 ′ ‐TTCTGCTGCTAATGTTCTTGACC‐3 ′
	Reverse 5 ′ ‐GGGATGAAGTATTGTGTCTTGGG‐3 ′

### ELISA

The chondrocytes’ culture supernatant was collected and operated according to the instructions of the Mouse VEGFA ELISA Kit (Beyotime, Shanghai, China). The absorbance at 450‐nm wavelength was measured with a microplate reader, and the expression of VEGFA of chondrocytes was calculated according to the standard curve.

### Western blotting

Mice chondrocyte cultures were extracted with RIPA lysis buffer containing protease inhibitors (Roche, West Sussex, UK). Proteins were resolved by 12% SDS/PAGE electrophoresis and transferred to a poly(vinylidene difluoride) membrane (Millipore, Billerica, MA, USA). The membrane was blocked with 5% nonfat milk in TBST buffer, probed with primary antibodies specific for phosphorylated p38 mitogen‐activated protein kinase (p‐p38 MAPK, 1 : 1000; CST, Danvers, MA, USA), p38 MAPK (1 : 1000; CST), p‐JNK (1 : 1000; CST) and JNK (1 : 1000; CST) at 4 °C overnight. β‐Actin (1 : 1000; Santa Cruz, Dallas, TX, USA) was applied to normalise the protein expression levels. Appropriate secondary antibodies were used to visualise the protein signals (1 : 5000; ZSGB‐BIO, Beijing, China). The signal was detected with chemiluminescence (Pierce, Rockford, IL, USA) according to the manufacturer’s protocols.

### Surgical mouse model of OA

Ten‐week‐old male C57BL/6J mice were purchased from HuaFukang Biotechnology Company (Beijing, China). DMM surgery was performed on the right knee joints of the 10‐week‐old male mice according to the procedure described in a previous study 23. The mice were first anaesthetised (1% pentobarbital sodium), the joint capsule was incised, and then the medial meniscotibial ligament was sectioned by using microsurgical scissors. As a control, sham operation was performed on the left knee joint with medial capsulotomy only, and the joints were categorised into the Sham group. Animals were randomly divided into the DMM and DMM + LIPUS groups (*n* = 7, each group), maintained in the animal facility (specific pathogen‐free) of Daping Hospital and allowed to move freely in the cages. All animal protocols were approved by the Institutional Animal Care and Use Committee of Daping Hospital (Chongqing, China, No: SYXK‐PLA‐2017‐0058).

### Animals treated with LIPUS

In order to avoid wound infection, we began to treat the animals with LIPUS on the third day after the DMM surgery. Mice were anaesthetised, the hair around the knee joint was shaved, and then a layer of coupling agent was applied between the LIPUS probe and mice joint (Fig. 1C,D). Mice underwent treatment with LIPUS at an average intensity of 30 mW·cm^−2^, frequency of 1.5 MHz, pulse repetition rate of 1 kHz and the on–off ratio of 20% for 20 min per day, as well as therapeutic parameters in clinic, once daily for 6 days as one cycle, which was continued for two cycles. We affixed the LIPUS device to the DMM group without electricity at the same time 11.

### Specimen preparation

Mice were sacrificed by CO_2_ inhalation at 8 and 12 weeks after the DMM surgery, knee joints were fixed in 4% paraformaldehyde, decalcified in 20% formic acid and embedded in paraffin. Serial sagittal sections were obtained across the entire joint by collecting 5‐µm sections. Sections were stained with Safranin O–Fast Green for histological analysis. The intervening sections were used for the immunohistochemical analysis.

### Immunohistochemical analysis

Knee joint sections were deparaffinised with xylene, and endogenous peroxidase activity was quenched by 3% H_2_O_2_, followed by antigen retrieval with 0.1% trypsinisation. Sections were then blocked with goat serum and incubated at 4 °C overnight with primary antibody followed by the appropriate biotinylated secondary antibody and horseradish peroxidase‐conjugated streptavidin–biotin staining. Immunoreactivity was visualised with a 3,3′‐diaminobenzidine tetrahydrochloride kit (ZSGB‐BIO) followed by counterstaining with methyl green. Primary antibodies against the following proteins were used: VEGFA (1 : 200; Abcam, Cambridge, MA, USA), collagen Π (1 : 200; Chondrex, Redmond, WA, USA), aggrecan (1 : 100; Abcam) and metalloproteinase‐13 (MMP‐13, 1 : 200; Abcam). The number of immunoreactive cells in the sections was counted using image‐pro plus 6.0 (Media Cybernetics, Rockville, MD, USA).

### Histologic assessment of articular cartilage degeneration

Histological changes in the medial tibial plateau (MTP) and medial femoral condyle (MFC) of the knee joints were scored on a scale of 0–6 according to the recommendations of the Osteoarthritis Research Society International (OARSI) 24. The maximum scores (the highest score in all slides) and summed scores (sum of the four highest scores in all slides) for the medial femora and medial tibiae were calculated separately to evaluate the severity of cartilage destruction. Scoring was carried out by three independent investigators, and all investigators were blinded to the allocation during experiments.

### Statistical analysis

Statistical analysis was performed using graphpad prism v.6.01 software (GraphPad Inc, La Jolla, CA, USA). Results were presented as mean ± SEM. Mean differences between two groups were analysed with the Student’s *t*‐test. Statistical analysis for multicomparisons was performed by one‐way analysis of variance (ANOVA). *P* values < 0.05 were considered statistically significant (*P < 0.05, **P < 0.01, ***P < 0.001).

## Results

### LIPUS directly reduces the expression of VEGFA and catabolic genes in IL‐1β‐treated mouse primary chondrocytes

**Figure 2:**
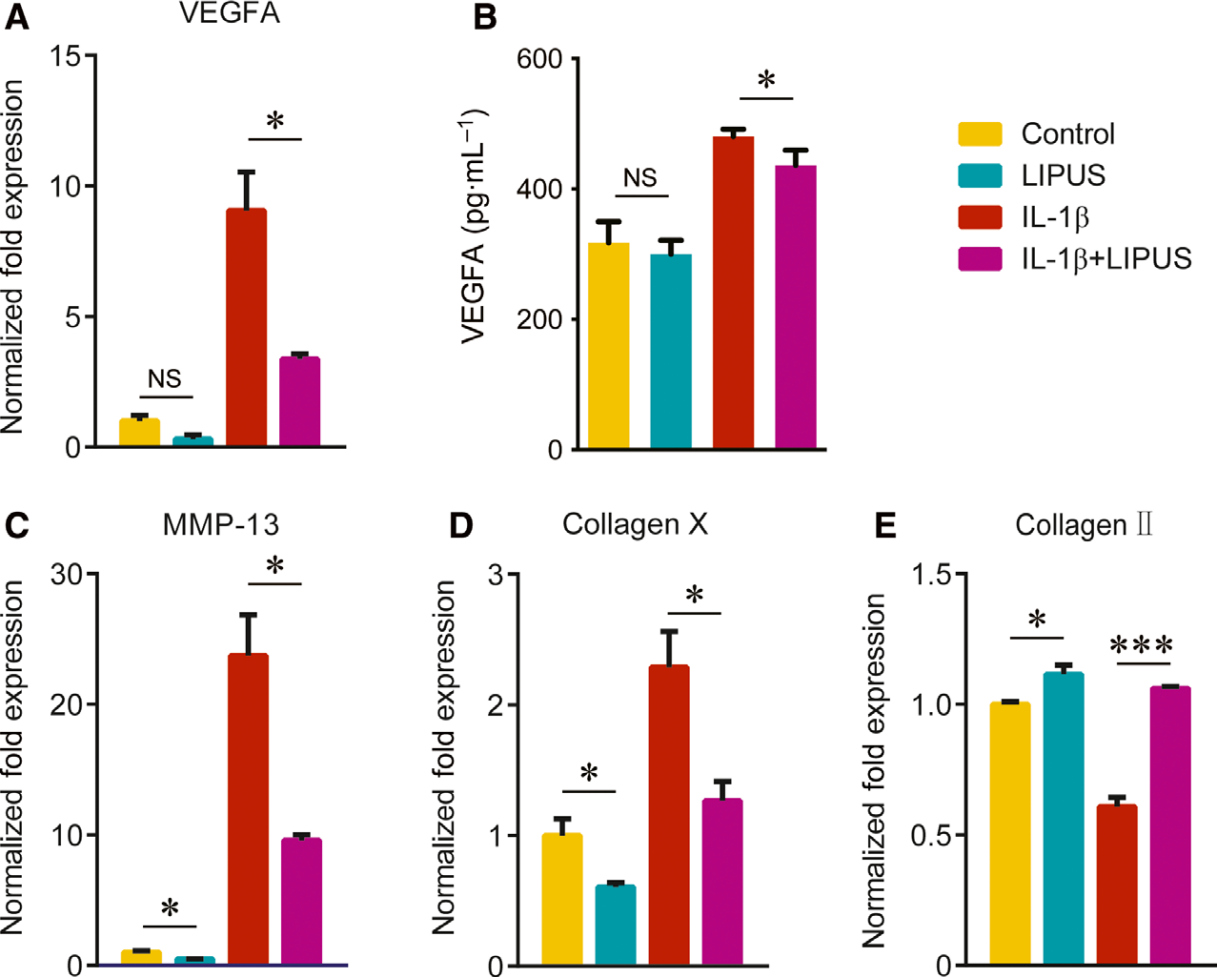
LIPUS regulates VEGFA and catabolic event‐related factor expression in primary chondrocytes. Mouse primary chondrocytes were incubated to 70–80% confluence, stimulated with 10 ng·mL^−1^ IL‐1β (PeproTech, Rocky Hill, NJ, USA) for another 24 h. Two hours before mRNA was collected, cells were treated with LIPUS for 20 min. qPCR showed the relative quantification of VEGFA (A), MMP‐13 (C), collagen X (D) and collagen Π (E) mRNA levels in the chondrocytes of each group. Twelve hours before the supernatant was collected, cells were stimulated with LIPUS. ELISA showed the protein level of VEGFA in the chondrocyte supernatant of each group (B). Statistical analyses were performed using Student’s *t*‐test. Data are expressed as the mean ± SEM of triplicate samples. NS, not significant, *P < 0.05, ***P < 0.001.

To clarify whether LIPUS directly regulates the expression of VEGFA in chondrocytes, we used mouse primary chondrocytes stimulated by IL‐1β to establish the OA model *in vitro*. IL‐1β has been well known to play a key role in the degradation of articular cartilage by inhibiting ECM synthesis and accelerating cartilage breakdown 25. Real‐time PCR and the ELISA results showed that LIPUS significantly alleviated the IL‐1β‐induced VEGFA expression at both mRNA (Fig. 2A) and protein (Fig. 2B) levels. However, there was no significant difference in normal chondrocytes with or without LIPUS treatment (Fig. 2A,B). Previous studies have demonstrated that VEGFA accelerates OA progression by promoting cartilage matrix degradation and chondrocyte hypertrophy 18. In mouse primary chondrocytes treated with IL‐1β, LIPUS significantly downregulated the levels of MMP‐13 and collagen X, as well as increased the expression of collagen Π (Fig. 2C–E). These results demonstrated that LIPUS directly reduced the expression of VEGFA and inhibited catabolic events of cartilage matrix in IL‐1β‐treated chondrocytes.

### LIPUS inhibits IL‐1β‐induced VEGFA expression by decreasing the phosphorylation of p38 MAPK

**Figure 3:**
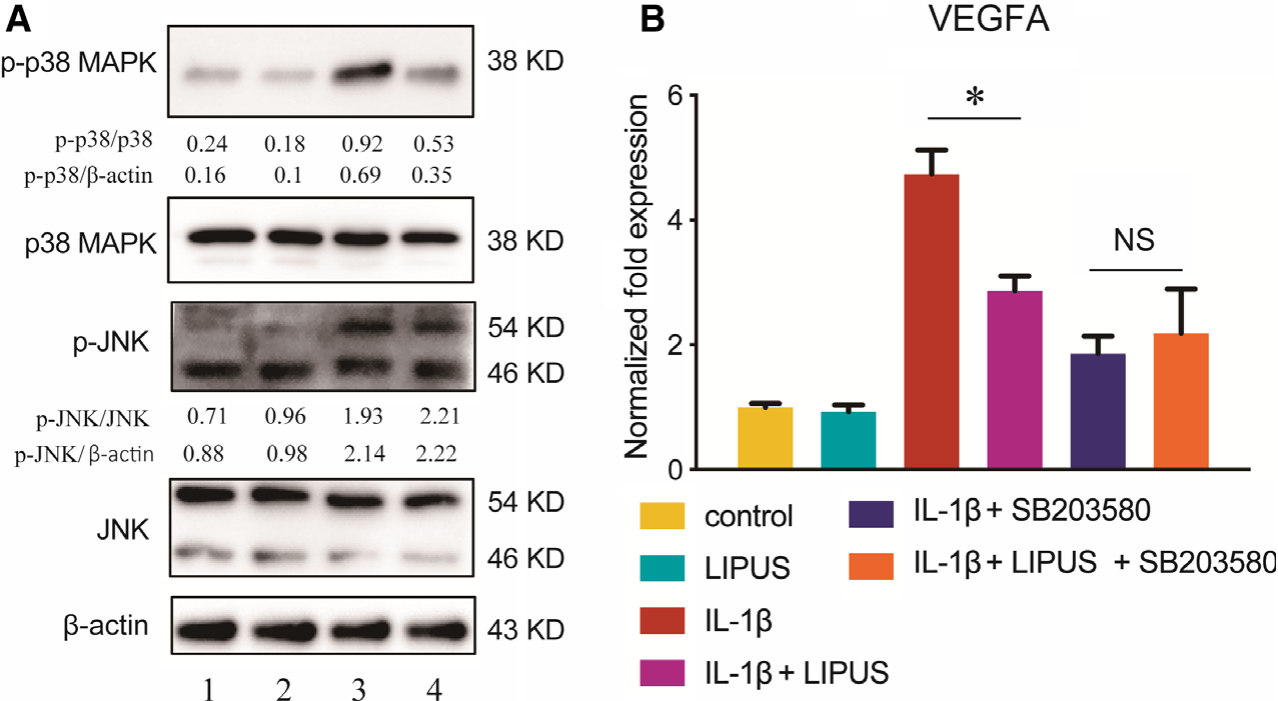
The p38 MAPK pathway participates in the inhibition effects of LIPUS on VEGFA. Mouse primary chondrocytes were incubated to 70–80% confluence, stimulated with 10 ng·mL^−1^ IL‐1β for another 24 h. Six hours before the cell lysates were collected, the cells were treated with LIPUS for 20 min. Cell lysates were analysed by western blotting using antibodies for phosphorylated and total p38 MAPK and JNK (A), 1: control group; 2: LIPUS group; 3: IL‐1β group; and 4: IL‐1β + LIPUS group. The signal intensities of p‐p38 MAPK/p38 MAPK, p‐p38 MAPK/β‐actin, p‐JNK/JNK and p‐JNK/β‐actin are presented. Chondrocytes were treated as before and pretreated with 10‐mm SB203580 (MedChemExpress, Princeton, NJ, USA) for 2 h before the LIPUS stimulation. mRNA was collected after LIPUS stimulation for 2 h. qPCR showed the relative quantification of VEGFA mRNA levels in the chondrocytes of each group (B). Statistical analyses were performed using Student’s *t*‐test. Data are expressed as the mean ± SEM (*n* = 3). NS, not significant, *P < 0.05.

Previous researches have shown that the abnormal activation of p38 MAPK and JNK signalling pathways is associated with increased VEGFA expression in osteoarthritic chondrocytes 26, 27. Therefore, through western blotting, we analysed phosphorylated and total protein levels of p38 MAPK and JNK in the total cell lysates of mouse primary chondrocytes cultured in the absence or presence of IL‐1β and LIPUS. We found that IL‐1β significantly increased the expression of p‐p38 MAPK and phosphorylated JNK (p‐JNK) compared with the control. Moreover, LIPUS attenuated IL‐1β‐upregulated p‐p38 MAPK protein levels of the primary chondrocytes (Fig. 3A), but it demonstrated no significant regulation of p‐JNK expression (Fig. 3A). These results indicated that LIPUS inhibits IL‐1β‐induced abnormal activation of the p38 MAPK signalling pathway.

To investigate whether pharmacological inhibition of p38 MAPK signalling could attenuate the downregulated VEGFA caused by LIPUS in IL‐1β‐treated chondrocytes, we pretreated chondrocytes with SB203580, a p38 MAPK inhibitor, which suppresses it by inhibiting the activity of phosphoinositide‐dependent kinase‐1. The qPCR results displayed that the presence of SB203580 negated the effects of LIPUS on VEGFA expression (Fig. 3B). This demonstrated that LIPUS abrogated IL‐1β‐induced VEGFA expression partially by regulating the p38 MAPK pathway in the articular chondrocytes.

### LIPUS decreases the expression of VEGFA and cartilage matrix loss *in vivo*

**Figure 4:**
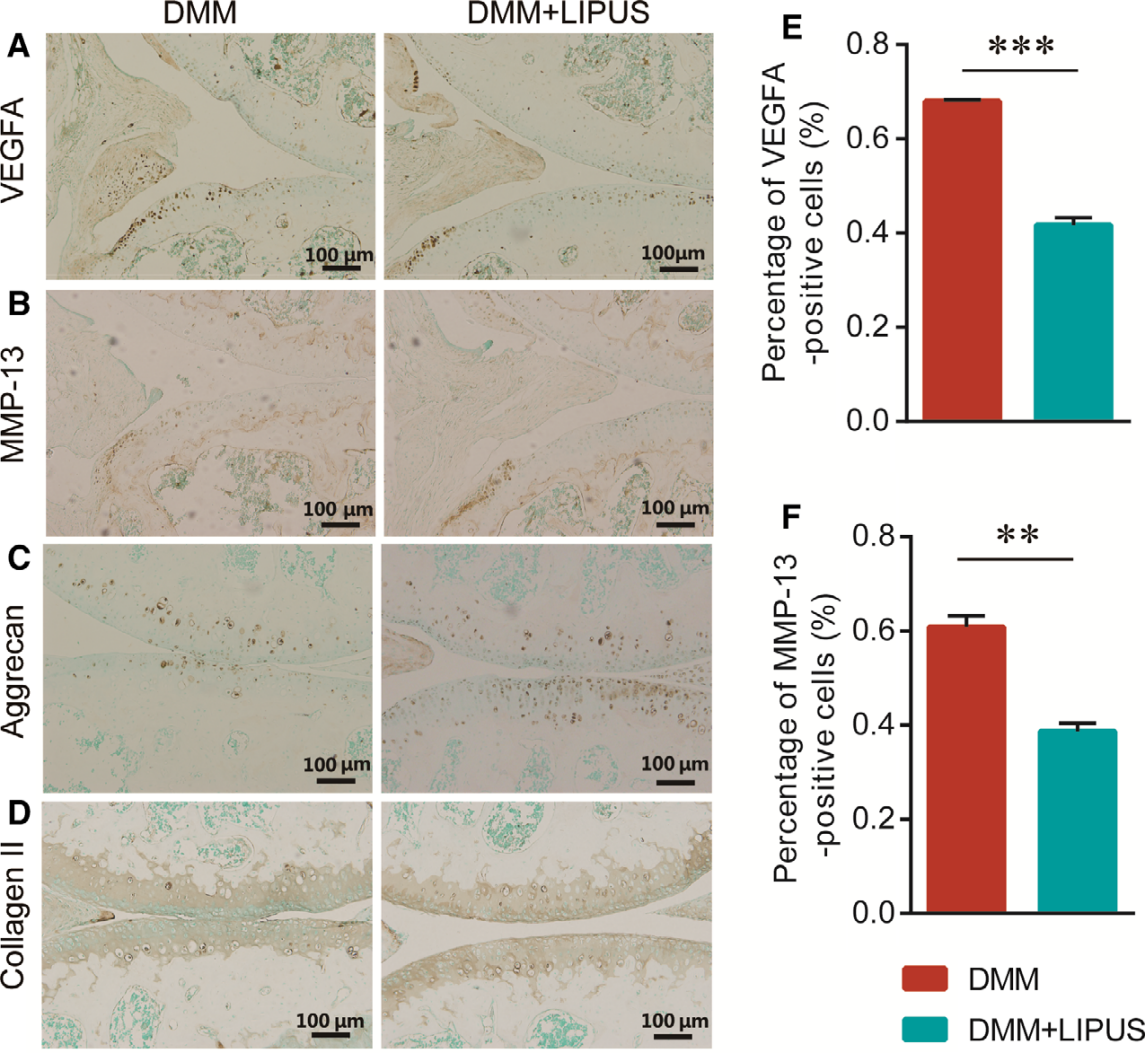
Effects of LIPUS on VEGFA and articular cartilage homeostasis in the mouse after DMM. The knee joints were harvested at 8 weeks after surgery and immunohistochemically stained for VEGFA (A), MMP‐13 (B), aggrecan (C) and collagen Π (D) expression in the DMM and DMM + LIPUS groups (scale bar: 100μm). The percentage of VEGFA‐positive cells (E) and MMP‐13‐positive cells (F) of the articular cartilage in each group. Statistical analyses were performed using Student’s *t*‐test. Data are expressed as the mean ± SEM (*n* = 5 mice per group). **P < 0.01, ***P < 0.001.

To investigate whether LIPUS modulated the expression of VEGFA *in vivo*, we performed DMM surgery on the right knee joint of 10‐week‐old male C57BL/6J mice, which is relevant to the actual state of human OA. After the DMM surgery, the mice received LIPUS treatment for 2 weeks and then were sacrificed at 8 weeks after DMM. We performed IHC staining to examine the expression of VEGFA, MMP13, aggrecan and type Π collagen (collagen Π). The results showed that LIPUS treatment significantly decreased the percentage of VEGFA‐positive cells in the articular cartilage (Fig. 4A,E). Furthermore, the IHC results showed that the LIPUS relieved loss of aggrecan and collagen Π as well as reduced the expression of MMP‐13, which is an essential cartilage matrix‐degrading enzyme (Fig. 4B–D,F). Collectively, these results suggested that LIPUS reduced the expression of VEGFA in the articular cartilage and alleviated the loss of cartilage matrix in the DMM‐induced OA model.

### LIPUS attenuates the cartilage degeneration in the DMM model

**Figure 5:**
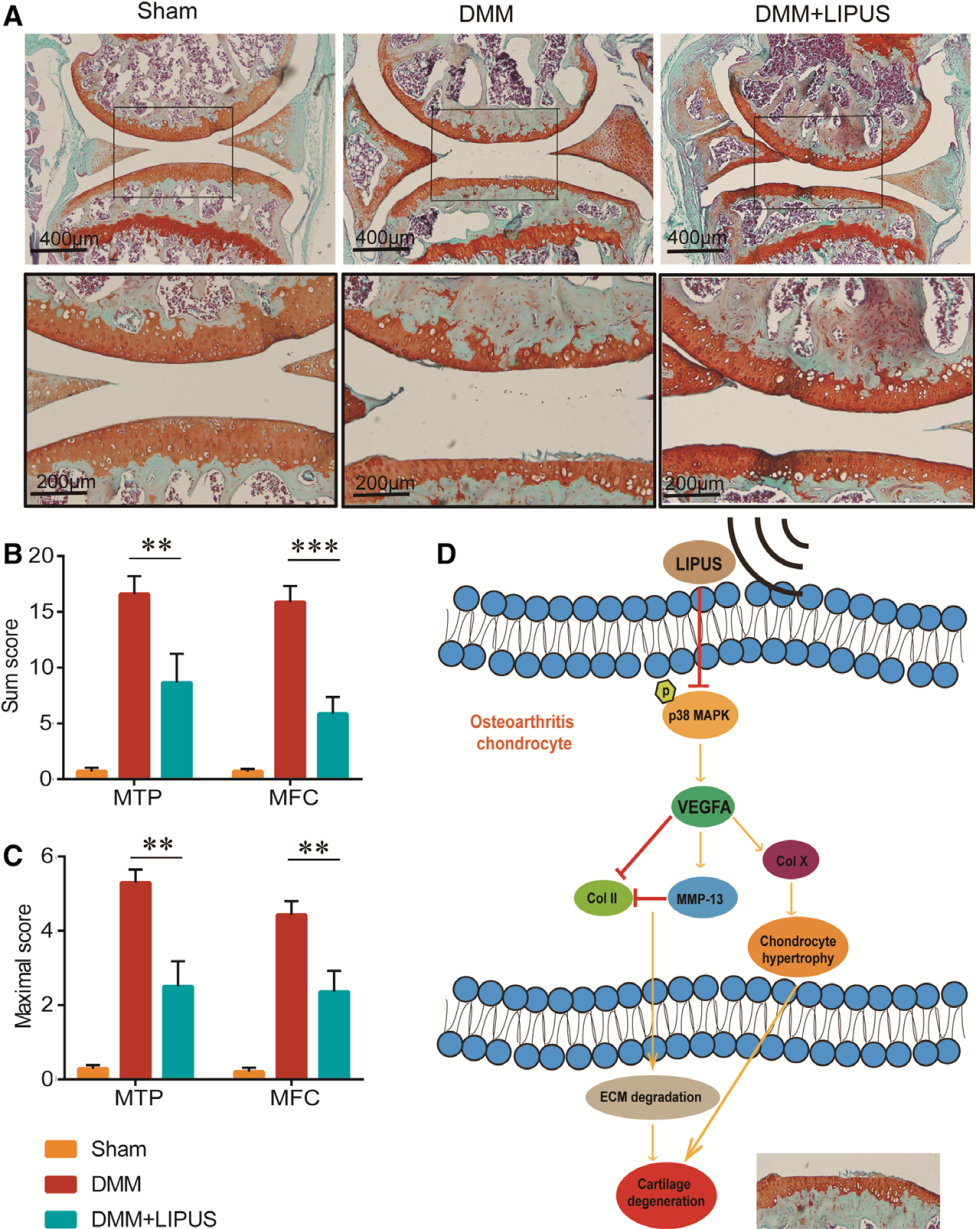
Effects of LIPUS on cartilage degeneration in articular cartilage. Mouse knee joints were harvested at 12 weeks after surgery and analysed histologically through Safranin O–Fast Green staining [scale bar, 400 μm (upper panels), 200 μm (lower panels)] (A). The joint degradation was evaluated histologically by the OARSI recommended scoring system. Sum (B) and maximal scores (C) were calculated for the MTP and MFC. LIPUS directly protects articular cartilage from degeneration by inhibiting VEGFA expression, and this effect is mainly exerted through the inhibition of p38 MAPK signalling (D). Statistical analyses were performed using Student’s *t*‐test. Data are expressed as the mean ± SEM (*n* = 7 mice per group). **P < 0.01, ***P < 0.001.

We further examined the therapeutic role of LIPUS in the development of OA. In the DMM model, Safranin O–Fast Green staining showed a significant reduction in hypertrophic chondrocytes, proteoglycan loss and articular cartilage degeneration in mice treated with LIPUS compared with control mice at 12 weeks after surgery (Fig. 5A). The OARSI histologic scoring system was applied to quantitatively analyse the cartilage degeneration in each group. The summed and maximal OARSI scores of the femurs and tibiae demonstrated that LIPUS‐treated mice had a significantly lower score than the DMM mice at 12 weeks after the surgery (Fig. 5B,C). These results suggested that LIPUS can significantly delay the progression of DMM‐induced knee OA in mice.

## Discussion

In this study, we explored a previously unrecognised effect of LIPUS on the inhibition of chondrocyte‐derived VEGFA in OA. We proved that LIPUS directly reduces the expression of VEGFA in IL‐1β‐treated mouse primary chondrocytes and downregulates VEGFA‐positive cells in the articular cartilage of the DMM mice model.

LIPUS, a noninvasive physical therapy, was recently used in clinical settings to relieve pain and improve the quality of life of patients with knee OA 9. However, the mechanisms involved in LIPUS‐treated OA are largely unknown. A previous study reported that LIPUS facilitated fracture healing by inducing VEGFA expression in periosteal cells and inducing local angiogenesis 15. Meanwhile, in a steroid‐associated osteonecrosis rat model, LIPUS promoted bone repair by increasing BMP2 rather than VEGFA expression in the femoral head 28. Besides, LIPUS facilitated bone–tendon junction repair through the upregulated VEGFA expression of chondrocytes and osteoblasts in woven bone 29. However, during rat meniscal healing, LIPUS promotes the migration and the VEGFA expression of meniscus cells in the outer meniscal region, but in the inner region with avascular tissue as articular cartilage, the modulation of VEGFA expression by LIPUS is still unknown 30. Therefore, LIPUS may regulate VEGFA expression in a cell‐intrinsic manner under different pathophysiological situations.

To date, numerous studies have suggested that VEGFA plays an important role in cartilage development and OA progression 17, 20, 31, 32, 33. Articular cartilage is a special and avascular tissue, which is composed of chondrocytes and ECM 34. VEGFA is barely expressed by healthy articular cartilage, whereas it acts as a promotive factor in the chain of events leading to OA 17. In knee OA patients, the expression of VEGFA in the articular cartilage, synovial fluid and synovium has been found to be significantly correlated with the grade of OA severity and the degree of pain 31. Moreover, intra‐articular administration of the anti‐VEGFA antibody or reduction in collagen Π lineage cell‐derived VEGFA attenuated the progression of surgically induced OA in rabbit or mice, respectively 20, 35. The above‐mentioned studies combined with our findings revealed that the therapeutic effects of LIPUS on OA may partially be mediated by downregulation of the expression of VEGFA in the articular cartilage.

Previous researches have shown that intra‐articular injection of VEGFA in the knee and temporomandibular joints of mice induced cartilage degeneration, which is correlated with subchondral bone sclerosis and increased expression of metalloproteinases and loss of cartilage matrix 18, 19. We have confirmed that LIPUS downregulated MMP‐13 expression and relieved loss of collagen Π in both the articular cartilage of mice which underwent DMM surgery and IL‐1β‐treated primary chondrocytes, which demonstrated that LIPUS provided anabolic effects on chondrocytes to alleviate articular cartilage degeneration. Consistent with our findings, the protective impacts of LIPUS on chondrocyte ECM also have been reported in rabbit and rat OA models 36, 37. Moreover, we also noticed that LIPUS inhibited the IL‐1β‐induced expression of collagen X, which is considered as the standard marker of chondrocyte hypertrophy. Previously, it was shown that VEGFA inhibition attenuated the expression of collagen X in chondrocytes both in a surgery‐induced rat OA model and in TNF‐α‐induced hypertrophic chondrocyte model 38. Therefore, we speculated that LIPUS ameliorated VEGFA‐mediated disorder in cartilage ECM metabolism and chondrocyte hypertrophy during OA development.

Evidences demonstrated that abnormal activation of p38 MAPK signalling pathway in OA chondrocytes was directly involved in the upregulation of VEGFA 26, 27. However, we found that LIPUS significantly inhibited the activation of p38 MAPK signalling in chondrocytes treated with IL‐1β. A previous study has suggested that the p38 MAPK is a major signalling molecule in LIPUS‐induced cartilage matrix maintenance in the rabbit OA model 39. We also showed that SB203580, a p38 MAPK inhibitor, negated the effects of LIPUS on VEGFA expression in IL‐1β‐treated chondrocytes. Our findings suggested that LIPUS reduced VEGFA expression in OA chondrocytes by inhibiting p38 MAPK signalling to maintain cartilage homeostasis during OA progression (Fig. 5D).

In summary, our findings highlight the novel effect of LIPUS on regulating the expression of osteoarthritic chondrocyte‐derived VEGFA. We found that LIPUS directly protects articular cartilage from degeneration by inhibiting VEGFA expression, and this effect is mainly exerted through the inhibition of p38 MAPK signalling. However, the exact mechanisms underlying this phenomenon warrant further study.

## Conflict of interest

The authors declare no conflict of interest.

## Author contributions

DQB, XLD, MTG and YZ conceived and designed the project; MTG, YZ, BL, BZ and JLH acquired the data; MTG, YZ and HBQ analysed and interpreted the data; MTG, YZ and QYT wrote the paper; all authors were involved in checking the paper and contributed to the preparation of the final manuscript. All authors read and approved the final manuscript.
